# Evaluation of the expression pattern and diagnostic value of PPARγ in malignant and benign primary bone tumors

**DOI:** 10.1186/s12891-022-05681-3

**Published:** 2022-08-03

**Authors:** Amir Reza Eghtedari, Mohammad Amin Vaezi, Banafsheh Safizadeh, Ghasem Ghasempour, Pegah Babaheidarian, Vahid Salimi, Masoumeh Tavakoli-Yaraki

**Affiliations:** 1 Department of Biochemistry, School of Medicine, Iran University of Medical Sciences, P.O. Box: 1449614535, Tehran, Iran; 2 Department of Pathology, School of Medicine, Iran University of Medical Sciences, Tehran, Iran; 3 Department of Virology, School of Public Health, Tehran University of Medical Sciences, Tehran, Iran

**Keywords:** PPARγ, Bone tumor, Osteosarcoma, Ewing sarcoma, GCT

## Abstract

**Purpose:**

The quantifiable description of PPARγ expression pattern beside mechanistic in-vitro evidence will provide insights into the involvement of this mediator in tumor pathogenesis. This study is focused on illuminating the PPARγ gene and protein expression pattern, its association with tumor deterioration and its diagnostic value in different types of primary bone tumors.

**Methods:**

The expression pattern of PPARγ was investigated in the 180 bone tissues including 90 bone tumor tissues and 90 non-cancerous bone tissues. The local PPARγ expression level was assessed using real-time qRT-PCR and the PPARγ protein expression pattern was measured using immunohistochemistry. The correlation of PPARγ expression level with patients’ clinic-pathological features, also the value of the variables in predicting PPARγ expression level in tumors and the value of PPARγ to discriminate tumor subtypes were assessed.

**Results:**

The mean PPARγ mRNA expression was significantly higher in bone tumors compared to healthy bone tissues, also the malignant tumors including osteosarcoma and Ewing sarcoma had the elevated level of PPARγ mRNA compared to GCT tumors. Consistently, the protein expression of PPARγ in the tumor site was significantly higher in the bone tumors and malignant tumors compared to non-cancerous and benign tumors, respectively. The PPARγ protein could predict malignant tumor features including tumor grade, metastasis and recurrence significantly. Moreover, PPARγ could potentially discriminate the patients from the controls also malignant tumors from benign tumors with significant sensitivity and specificity.

**Conclusions:**

PPARγ might be involved in primary bone tumor pathogenesis and determining its molecular mechanism regarding bone cancer pathogenesis is of grave importance.

## Introduction

Primary bone cancers account for heterogeneous sarcomas with a mesenchymal origin that is associated with significant morbidity and mortality and reduced overall survival [1]. Osteosarcoma and Ewing sarcoma are among the most common malignant primary bone tumors that initially induce pain and swelling in patients [2]. Osteosarcoma is characterized by local pain, localized swelling and limited joint movement that is more frequent in adolescence [3]. However, Ewing sarcoma usually characterized by tiredness, high temperature, unintentional weight loss and EWS-ETS family of gene fusions that might provide therapeutic opportunities to its better treatment [4]. Despite the positive effect of chemotherapy to improve the bone cancer outcome, surgical resection is still the most confident curative procedure, however, 25% of patients develop metastasis lesions after receiving treatments [5]. The diagnosis of primary bone cancers is relying on invasive approaches such as biopsy, that making it necessary to find promising molecular targets and biomarkers with significant diagnostic and predictive value [6]. Peroxisome proliferator-activated receptors (PPARs) are ligand-activated transcription factors consisting of three isotypes, PPAR *α*, *γ*, and $\delta$, that belong to the nuclear hormone receptor superfamily [7]. PPARs account as master metabolic regulators and lipid sensors which are implicated in many aspects of energy homeostasis, cell growth and cell fate [8]. PPARα is involved in the activation of fatty acid catabolism in liver cells, while PPARδ is implicated in fatty acid oxidation [9]. PPARγ plays a pivotal role in glucose metabolism, adipocyte differentiation, cell cycle regulation, lipid storage and inflammation [10]. Aside from the established properties of PPARγ, the relevance of PPARγ to the regulation and differentiation of cancer cell growth is increasingly recognized. The tight linkage between PPARγ and cancer has attributed to the multi functions of PPAR*γ* in metabolic reprogramming of cancer cells, tumor cell-associated secretions, tumor microenvironment and adaptations also immune response [11], however, the oncogenic or tumor suppressive role of PPARγ is controversial and dependent on the tumor cell type, origin, individual-specific manner and a dose concentration [12]. In support of the tumoricidal effect of PPARγ, it was shown that its activation is associated with overexpression of vascular endothelial growth factor A (VEGF-A) and vimentin, as the major component of cell migration and angiogenesis [13]. While multiple lines of evidence highlighted the tumor suppressive role of PPARγ in regulating cancer cell growth and PPARγ agonists induced different types of programmed cell death pathways in cancer cells [14]. In particular, regrading bone tissue, PPARγ is involved in skeletal remodeling and regulates both mesenchymal and hematopoietic lineages cells thus playing dual role in bone homeostasis [15]. In accordance, the increase in osteoblast number and bone mass and formation were detected in PPAR*γ* heterozygous mice [16]. In addition, PPAR*γ* inhibition by Wnt signaling mediators resulted in elevated osteoblastogenesis through reducing adipogenesis [17]. Also, it was shown that PPARγ activation is required for caspase-3 and caspase-9 activity, reactive oxygen species (ROS) generation and apoptosis-induced by oridonin in human osteosarcoma cells [18].Accordingly, PPARγ and retinoid X receptor (RXR) overexpression induced apoptosis and suppress the proliferation of osteosarcoma cells possibly through promoting osteoblastic terminal differentiation [14]. The full understanding of the mechanisms underlying these complications remains unknown, and identifying the relevance of PPARγ, as a critical lipid metabolism regulator in primary bone cancer pathogenesis may enhance our understanding of the putative mechanisms underlying bone tumor growth also may introduce a more effective target for bone tumor therapeutic purposes. This study is designed to determine the gene and protein expression pattern of PPAR*γ* and its association with tumor severity, metastasis, recurrence in different types of primary bone tumors and bone normal tissues.

## Materials and methods

### Patients and sample collection

A total number of 180 bone tissues (90 tumor tissues and 90 tumor margins) were enrolled in the current study with local ethical approval and informed consent. This study was performed based on the guidelines of Helsinki Declaration [19]. The pair of tumor and margin tissue was taken from each patient during surgical resection at the Shafa Orthopedic Hospital and the collection protocol and the sample collection and storage protocol was performed according to our previous study [20]. Three types of primary bone tumors including osteosarcoma, Ewing Sarcoma and Giant Cell Tumor (GCT) were included in the current study and the clinic-pathological features of patients is shown in Table 1. As illustrated in Table 1, the equal number of patients with osteosarcoma, Ewing Sarcoma and GCT was enrolled in the study and the majority of participants had no history of a particular disease. Regarding age distribution, 63.3%, 33.3% and 46.7% of patients with osteosarcoma, Ewing Sarcoma and GCT were over 30 years of age, respectively. Also, 53.3%, 40% and 56.7% of patients with osteosarcoma, Ewing Sarcoma and GCT were male, respectively. The tumor size in 46.7%, 40% and 13.3% of patients with osteosarcoma, Ewing Sarcoma, and GCT was more than 10 cm. Notably, 63.3% and 66.7% of osteosarcoma and Ewing Sarcoma tumors were high-grade. In the current study, 56.7% of patients with osteosarcoma and Ewing Sarcoma received chemotherapy treatment before the surgery; while none of patients with GCT received chemotherapy before surgical resection. Additionally, the chemotherapy protocol for osteosarcoma patients was the standard combination of doxorubicin, cisplatin, and methotrexate and the chemotherapy protocol for Ewing Sarcoma patients was the combination of vincristine, cyclophosphamide, and doxorubicin and patients with the chemotherapy period of 10 weeks were included. Also, 30% of osteosarcoma and Ewing Sarcoma tumors in the current study were metastatic and 26.7% and 23.3% of osteosarcoma and Ewing Sarcoma tumors were recurrent tumors. The Laboratory findings of patients are summarized in the Table 2.

**Table 1:** The clinic- pathological features of patients with bone tumors

		Malignant bone tumor		Benign bone tumor
Demographic features	Groups	Osteosarcoma (*N* = 30)	Ewing Sarcoma (*N* = 30)	GCT (*N* = 30)
Age	30	11(36.6%)	20(66.7%)	16(53.3%)
	30	19(63.3%)	10(33.3%)	14(46.7%)
Gender	Male	16(53.3%)	12(40%)	17(56.7%)
	Female	14(46.7%)	18(60%)	13(43.3%)
Tumor size (cm)	10	16(53.3%)	18(60%)	26(86.7%)
	10	14(46.7%)	12(40%)	4(13.3%)
Tumor grade	Low (grade I/II)	11(36.7%)	10(33.3%)	30(100%)
	High (grade III)	19(63.3%)	20(66.7%)	0(0%)
History of particular disease	Positive	7(23.3%)	4(13.3%)	4(13.3%)
	Negative	23(76.7%)	26(86.7%)	26(86.7%)
Chemotherapy	Positive	17(56.7%)	17(56.7%)	0
	Negative	13(43.3%)	13(43.3%)	30(100%)
Metastasis	Yes	9(30%)	9(30%)	0
	No	21(70%)	21(70%)	30(100%)
Tumor recurrence	Yes	8(26.7%)	7(23.3%)	0
	No	22(73.3%)	23(76.7%)	v

**Table 2:** Frequency distribution of individuals based on biochemical and laboratory information

Parameters	Range	Numbers	(%)	Mean ± std
Fasting Blood Sugar (mg/dl)	Normal: 70–100	42	48.84	90.36 ± 7.06
	Low: <70	0	0	………………
	Border line: 100–120	26	30.23	108.11 ± 4.14
	High: ≥120	18	20.93	132.54 ± 14.13
SGPT	Normal: Male < 41	36	43.90	16.58 ± 6.79
	Female < 31	36	43.90	15.94 ± 6.22
	High: Male ≥ 41	3	3.66	57.33 ± 16.65
	Female ≥ 31	7	8.54	52.29 ± 43.12
SGOT (mg/dl)	Normal: Male < 38	37	45.12	19.51 ± 6.11
	Female < 32	34	41.46	19.53 ± 5.00
	High: Male ≥ 38	2	2.44	57.50 ± 19.56
	Female ≥ 32	9	10.98	48.00 ± 19.13
WBC (*1000/mm^3^)	Normal range: 4–10	82	92.14	6.98 ± 1.47
	Low: > 4	1	1.12	3.40 ± 0
	High: ≥10	6	6.74	12.58 ± 2.69
HB	Normal range: 14–18	32	36.36	15.37 ± 0.97
	Low: ≤14	54	61.36	12.21 ± 1.53
	High: ≥18	2	2.28	18.15 ± 0.21
RBC (*1000/mm^3^)	Normal range: 4.5–6.2	51	57.31	5.13 ± 0.36
	Low: ≤4.5	35	39.32	3.97 ± 0.51
	High: >6.2	3	3.37	6.30 ± 0.09
ESR (mm/hr)	Normal: Male: <15	31	35.23	4.64 ± 2.98
	Female: <20	32	36.36	6.34 ± 5.17
	High: Male ≥ 15	12	13.64	43.75 ± 26.96
	Female ≥ 20	13	14.77	73.31 ± 43.50

### RNA extraction, cDNA synthesis, and RearealmtimeR

The quantitative real-time PCR was applied to evaluate the gene expression level of PPARγ in bone tumor types. In this regards, the tumor and adjacent noncancerous bone tissue was used for RNA extraction using Trizol (Invitrogen, Grand Island, USA) based on the manufacturer’s instructions. To lysis bone tumor and normal tissues, 700µL of Trizol lysis reagent was used that followed by subsequent phase separation using chloroform. The isopropanol was used to mix with aqueous phase after separation that helped to extract RNA. The quantity of the extracted RNA was evaluated by Nanodrop spectrophotometer (Nanodrop Technologies) and to indicate the RNA integrity and purity, RNA was electrophoresed using 1% agarose gel. The PrimeScript First Strand cDNA Synthesis Kit (Takara, Japan) was applied for cDNA synthesis based on the manufacturer’s instructions. To evaluate the PPARγ expression level, The SYBR Premix Ex Taq II (Takara, Japan) was used that was implemented in Applied Biosystems Step One Plus, Real-time system (Applied Biosystems, USA). The specific primers were designed for PPARγ and β-actin, as endogenous housekeeping gene. The sequence of primers was as: PPARγ (forward primer): *5’– CGGTTTCAGAAATGCCTTGC − 3’* PPARγ (reverse primer): *5’-* TCAGCTGGTCGATATCACTG *− 3’* (Tm = 58) and β-actin forward primer: *5’-GAT CTC CTT CTG CAT CCT GT-3’*, β-actin reverse primer: *5’-TGG GCA TCC ACG AAA CTA C- 3’* (Tm = 57). The melting curve analysis was considered to evaluate primer’s specificity and the amplified products. The running PCR protocol was as 1 cycle at 95 °C for 5 min, 40 cycles at 95 °C for 5 s, 55 °C for 20 s and 60 °C for 35 s. 1% agarose gel electrophoresis was used to measure and evaluate the PCR products and the comparative CT (2^−ΔCt^) approach ( ΔCt represents the subtract of Ct of PPARγ from the Ct of the endogenous gene (β -actin)) was used to analyze the PPARγ gene expression.

### Tissue histopathology and immunohistochemically staining of PPARγ

The Hematoxylin and eosin (H&E) histological staining was performed based on the previously described protocol [21]. Briefly, tissue sections were dehydrated by alcohol (for 5 min), washed and stained with Harris’s hematoxylin (for 10 min). The differentiation of tissues in acid alcohol and incubation in lithium carbonate were done for 5 min. The staining with eosin for 15 s and dehydrating with alcohol and xylene were followed afterward. To evaluate the PPARγ protein level in tumor tissues of primary bone cancers, the level of PPARγ was evaluated using immunohistochemistry. Based on the protocol which was applied in our previous study [20], bone tissues were fixed and incubated in 4% paraformaldehyde and 20% sucrose and the frozen tissue blocks were prepared using Optimal Cutting Temperature (OCT) embedding medium. 10% normal serum with 1% BSA in TBS for 2 h at room temperature was applied for blocking and following appropriate washing, the endogenous peroxidase was inhibited using 1ml H2O2 and 9 ml ddH2o for 10 min. The slides were probed by PPARγ primary antibody (Abcam, USA) and following appropriate washing and incubation, slides were exposed to 1 µl 3′-diaminobenzidine (DAB) chromogen and 20 µl DAB substrate for 1 min. The anti-rabbit IgG HRP-conjugated secondary antibody (Abcam, USA) was used to visualize binding of the PPARγ primary antibody. The slides were examined by a pathologist after the staining process was completed and the staining intensity of PPARγ was quantified using Image J and reported as the percentage of positive reactivity [22]. Accordingly, from each sample several images were taken and the images were converted to the black and white images with a software. To evaluate the percentages of cells, the threshold set up was performed. Threshold adjustment was conducted according to the removal of background signals and without eliminating true signals. Then, the selected threshold was used to analyze all IHC images [22]. The process performed in a blinded manner and IHC images were evaluated at least three times.

### Statistical analysis

All data were assessed for normal distribution using the Kolmogorov-Smirnov analysis and side-to-side comparisons were conducted using the parametric unpaired t-test and nonparametric Mann–Whitney U test for multiple comparisons of PPARγ expression between tumor and margin, malignant and benign tumors, malignant and benign tumor subgroups and their matched normal margins also malignant tumors with different tumor features (chemotherapy history, response to therapy, grade, metastasis status and recurrence). To calculate the value of PPARγ gene and protein expression to discriminate tumor and normal tissue, the receiver operating characteristic (ROC) curves and calculation of area under the curve (AUC) were applied. The various cut- off points of PPARγ gene and protein level was evaluated for the sensitivity and specificity and the optimal cut-off value was determined based on the Youden index [23]. The optimal cut-off value indicates the PPARγ level of expression that has the maximum sensitivity and specificity to discriminate between two groups. The enumeration data are presented as percentages in the relevant tables and to determine the correlation of PPARγ gene and protein expression with patients’ age, tumor size and tissue gene expression, and protein level, Spearman correlation coefficient test was applied. The value of tumor different features in predicting PPARγ expression level was assessed using logistic regression analysis. The Graph Pad Prism version 6 (Graph Pad Software, San Diego California) and Statistical Package for Social Science (SPSS v.16) were used for the calculation of all statistics. *P* values < 0.05 (two-sided) were considered statistically significant.

## Results

### The PPARγ gene expression level in different types of primary bone tumors

**Fig. 1:**
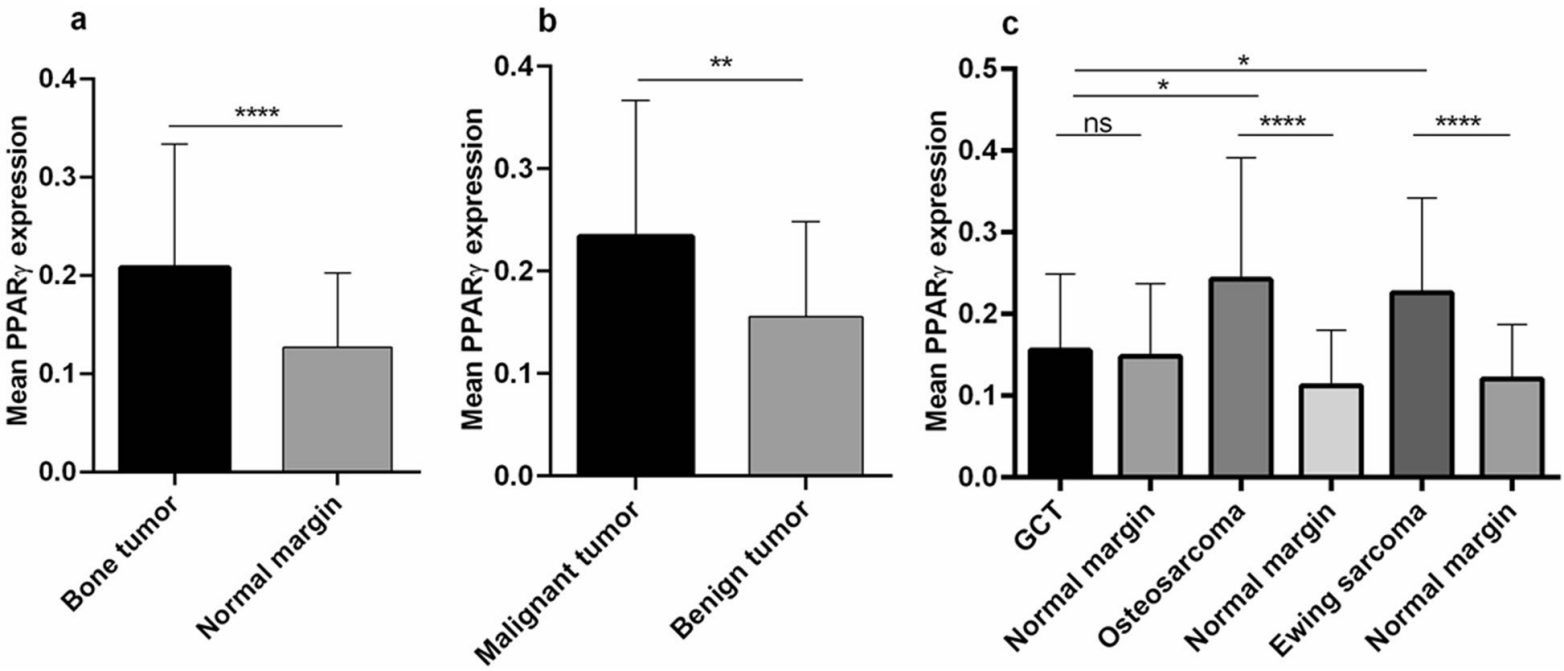
The assessed PPARγ mRNA expression levels in primary bone tumors. The mRNA expression level was evaluated in osteosarcoma, Ewing Sarcoma and GCT tumors. The mRNA expression level of PPARγ was increased in bone tumor (**a**) versus tumor margins also in malignant tumors versus benign tumors (**b**). The elevated level of PPARγ was detected in osteosarcoma and Ewing Sarcoma compared to their paired non-cancerous tissues (**c**). The statistical differences between groups are shown as asterisk (*= *P* < 0.05, **= *P* < 0.01, ****= *P* < 0.0001)

**Fig. 2:**
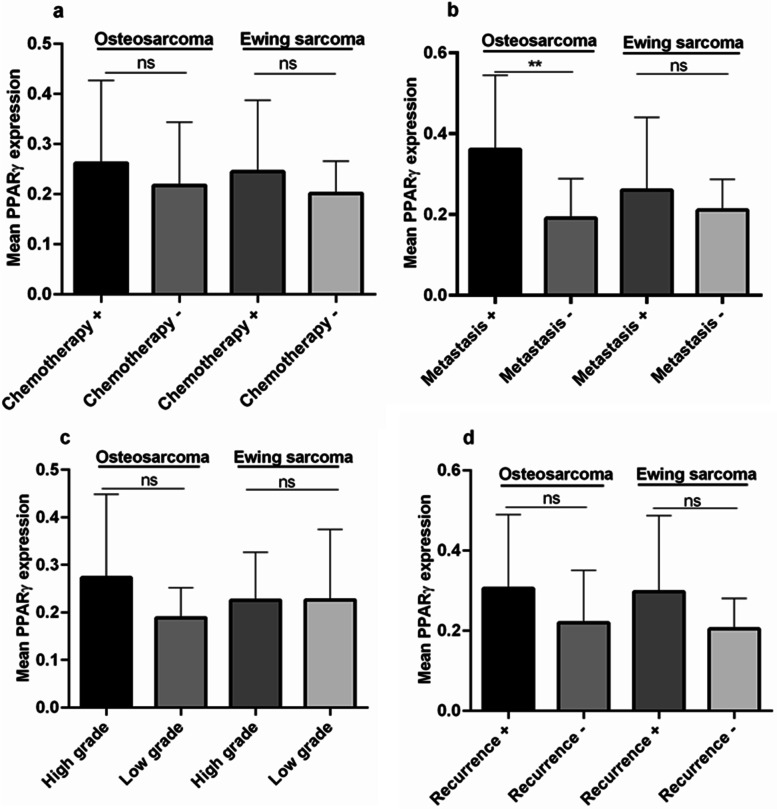
The assessed PPARγ mRNA expression levels in malignant tumor for the different clinic-pathological variables. The PPARγ mRNA expression level was evaluated in tumor tissue of osteosarcoma and Ewing Sarcoma patients with chemotherapy status (**a**), metastasis (**b**), tumor grade (**c**) and tumor recurrence (**d**). The type of tumor is indicated above the columns. The statistical differences between groups are shown as asterisk (**= *P* < 0.01) and (ns) indicates non-specific

As shown in Fig. 1a, the PPARγ mRNA expression level in bone tumor tissue was significantly higher than its expression level in matched noncancerous bone tissues (*P* < 0.0001). The mean and standard deviation (std) of PPARγ mRNA level in tumor and tumor margin groups was 0.21 ± 0.13 and 0.13 ± 0.08, respectively, indicating 1.6-fold increase in PPARγ mRNA level in bone tumors. Comparing the malignant and benign bone tumors, showed a significant increase in the PPARγ mRNA level in malignant (0.23 ± 0.13) vs. benign (0.16 ± 0.09) bone tumors (*P* = 0.0049) (Fig. 1b). To be specific, no significant difference was observed in the PPARγ mRNA level in GCT (0.16 ± 0.09) tumors compared to the matched normal tumor margins (0.15 ± 0.09) (Fig. 1c); while osteosarcoma tumors expressed significantly higher level of PPARγ compared to normal bone tissues (*P* = 0.0039). The mean PPARγ mRNA level in osteosarcoma tumors and tumor margin group was 0.24 ± 0.15 and 0.11 ± 0.07, respectively. A similar pattern of PPARγ expression level was observed in Ewing Sarcoma tumors compared to normal tissue that showed a significant elevation in tumor tissues (0.23 ± 0.12) compared to normal bone tissues (0.12 ± 0.07) (*P* = < 0.0001) (Fig. 1c). The different between PPARγ mRNA level in GCT tumors compared to osteosarcoma (*P* = 0.006) and Ewing Sarcoma tumors (*P* = 0.0107) was statistically significant (Fig. 1c). As shown in Fig. 2, the PPARγ mRNA expression levels in tumor tissue were compared as a matter of different tumor features. The PPARγ expression in osteosarcoma and Ewing Sarcoma tumors patients undergoing chemotherapy had no significant difference compared to untreated cases (Fig. 2a). Moreover, osteosarcoma patients suffering from metastasis (0.36 ± 0.18) expressed higher level of PPARγ mRNA level compared to non-metastatic patients (0.19 ± 0.1) (*P* = 0.0051); while the difference between Ewing Sarcoma metastatic and non-metastatic tumors was not significant (Fig. 2b). Although the osteosarcoma patients with high grade tumor tended to express higher PPARγ mRNA level, no significant difference was observed between high and low grade tumors in osteosarcoma and Ewing Sarcoma patients (Fig. 2c). The PPARγ mRNA level in recurrent osteosarcoma (0.31 ± 0.18) and Ewing Sarcoma (0.30 ± 0.19) tumors was increased compared to the non-recurrent osteosarcoma (0.22 ± 0.13) and Ewing Sarcoma (0.20 ± 0.08) tumors; while it was not statistically significant (Fig. 2d).

### The PPARγ protein expression level in different types of primary bone tumors

**Fig. 3:**
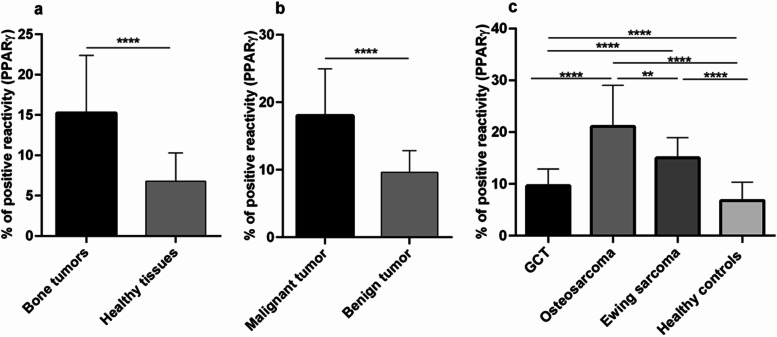
The assessed PPARγ protein expression in primary bone tumors. The PPARγ protein expression level was evaluated in different bone tumor tissue and non-cancerous paired margins. The enhanced level of PPARγ was detected in bone tissues compared to healthy tissues (**a**), and malignant tumors compared to benign tumors (**b**). The osteosarcoma and Ewing Sarcoma tumors showed higher PPARγ expression level compared to GCT tumors; while GCT tumors showed over expression of PPARγ compared to healthy bone tissues (**c**). The higher PPARγ expression was detected in osteosarcoma and Ewing Sarcoma tumors compared to healthy bone tissues, also osteosarcoma tumors showed the highest expression compared to other tumors (**c**). The statistical differences between groups are shown as asterisk (**= *P* < 0.01, ****= *P* < 0.0001)

**Fig. 4:**
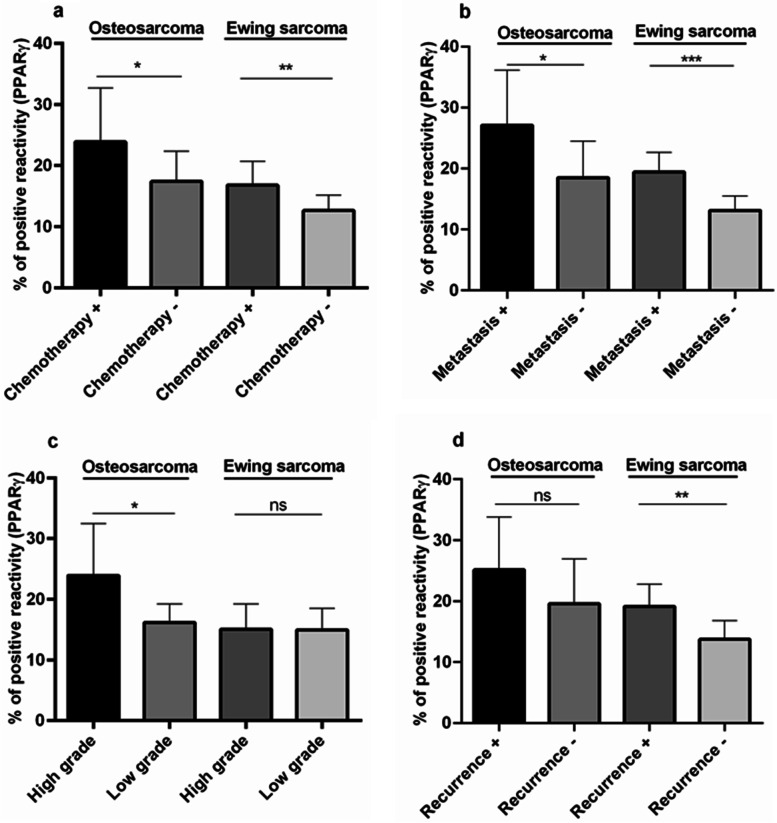
The assessed PPARγ protein expression levels in tumor tissue of malignant bone tumors for the different clinic-pathological variables. The PPARγ protein level was enhanced in chemotherapy-received osteosarcoma and Ewing Sarcoma tumors compared to chemotherapy negative tumors (**a**). The elevated PPARγ was detected in metastatic tumors compared to non-metastatic tumors in both groups (**b**). The high grade osteosarcoma tumors expressed higher PPARγ protein level compared to low grade tumors (**c**) and PPARγ protein level was increased in recurrent tumors compared to non-recurrent groups in both groups (**d**). The statistical differences between groups are shown as asterisk (*= *P* < 0.05, **= *P* < 0.01, ***= *P* < 0.001)

**Fig. 5:**
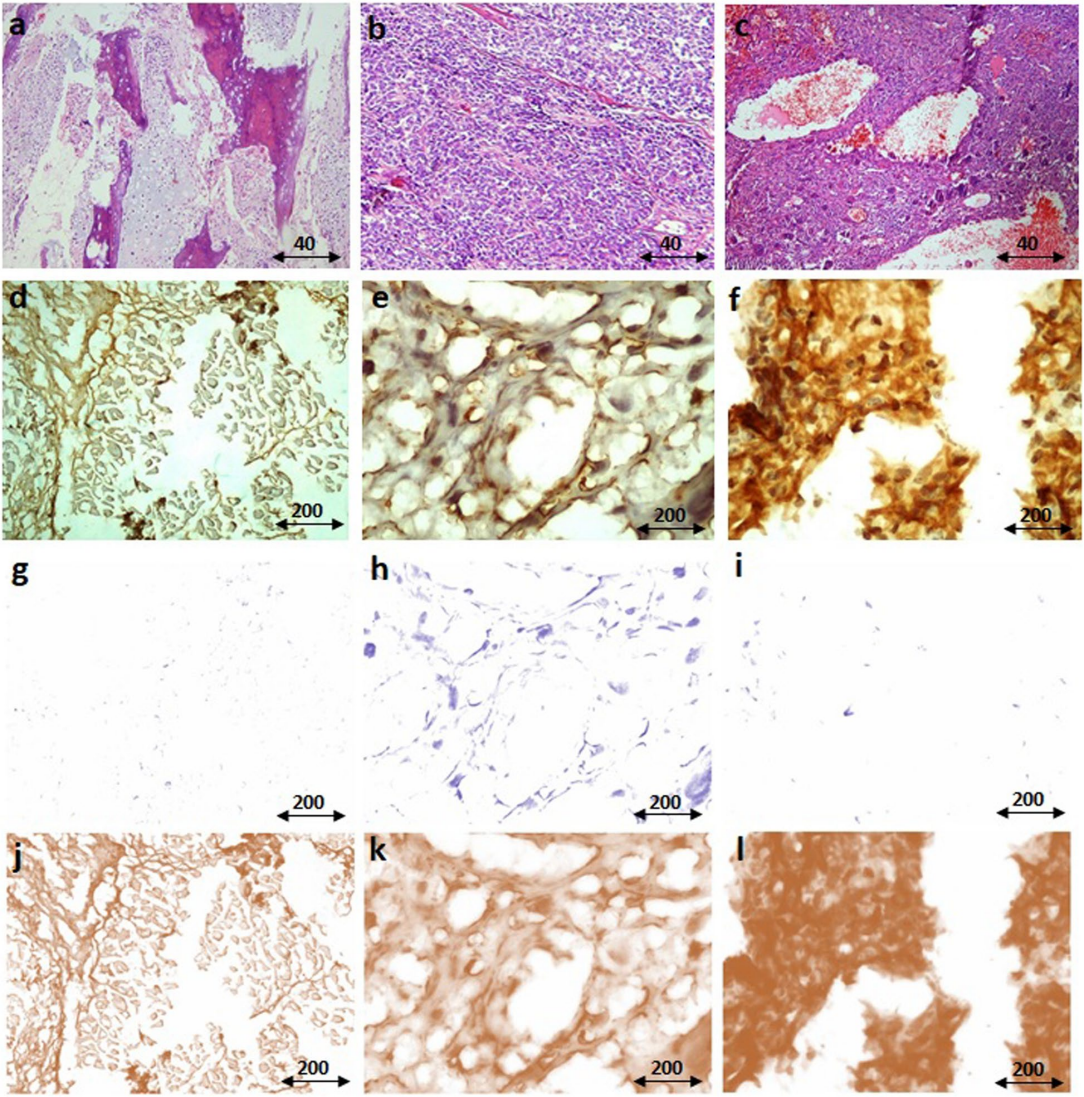
Immunohistochemistry staining of PPARγ protein in primary bone tumors. The hematoxylin and eosin (H&E) staining (**a**-**c**) and immunohistochemistry of PPARγ (**d**-**f**) in bone tumor tissues were assessed. The representative images of H&E staining of the osteosarcoma tumor tissue (**a**), Ewing sarcoma (**b**) and GCT (**c**) are shown. The negative immune-reactivity of PPARγ is shown in (**d**), the weak intensity of PPARγ (< 10% immune-reactivity) is shown in (**e**) and the strong intensity of PPARγ (> 20% immune-reactivity) is shown in (**f**). The cytoplasmic expression of PPARγ is shown in (**e**) and (**f**) that represents Ewing sarcoma and osteosarcoma tumor tissues, respectively. The separated immunohistochemistry images into staining for the nuclei and DAB-positive area is shown (**g**-**l**). The staining for the nuclei is illustrated in g-i as (**g**): negative staining, (**h**): weak intensity, and (**i**): strong intensity. The DAB staining for target PPARγ protein expression is shown in **j**-**l** as (**j**): negative intensity, (**k**): weak intensity, and (**l**): strong intensity. The analysis was followed the Crowe’s method [22]. The scale of magnification for (**a**-**c**) is 40 and for (**d**-**l**) is 200

The protein level of PPARγ is shown in Fig. 3 and the staining intensity of PPARγ is assessed as the percentage of positive reactivity. Based on data, the locally expression of PPARγ protein is increased in bone tumor tissues compared to non-cancerous tumor margins (*P* < 0.0001) (Fig. 3a). Comparison of malignant bone tumors with benign bone tumors revealed that the PPARγ protein expression in malignant tumors increased than the benign counterparts (*P* < 0.0001) (Fig. 3b). In specific, GCT tumors expressed a significant lower level of PPARγ protein compared to osteosarcoma (*P* < 0.0001) and Ewing Sarcoma tumors (*P* < 0.0001); while the protein level of PPARγ protein was significantly higher in osteosarcoma tumors compared to Ewing Sarcoma tumors (*P* = 0.001). Furthermore, the PPARγ protein level was found to be expressed more in osteosarcoma (*P* < 0.0001), Ewing Sarcoma tumors (*P* < 0.0001) and GCT (*P* < 0.0001) tumors compared to normal bone tissues (Fig. 3c). Interestingly, chemotherapy-received osteosarcoma (*P* = 0.039) and Ewing Sarcoma (*P* = 0.004) tumors illustrated higher level of PPARγ protein to the tumors without any history of chemotherapy, respectively (Fig. 4a). As shown in Fig. 4b, metastatic osteosarcoma (*P* = 0.031) and Ewing Sarcoma (*P* = 0.0002) tumors expressed significantly higher level of PPARγ protein compared to non-metastatic tumors (Fig. 4b). Despite of significant elevation in the PPARγ protein in high grade osteosarcoma tumors (*P* = 0.012), no specific difference was observed between low and high grade Ewing Sarcoma tumors (Fig. 4c). The PPARγ protein level was expressed non-significantly in recurrent osteosarcoma tumors; while significantly in recurrent Ewing Sarcoma tumors (*P* = 0.008) compared to tumors without recurrence in each group (Fig. 4d). The representative images of PPARγ protein immunohistochemistry staining in primary bone tumor tissues are illustrated in Fig. 5.

### The association of PPARγ expression with bone cancer patients with different demographic features

As shown in Table 3, according to the obtained regression model, expression of PPARγ protein expression in tumor site can be effective in predicting malignancy of bone tumors (OR = 1.59, CI = 1.28–1.98, *P*-value < 0.0001); the OR was not statically significant for PPARγ gene expression and tumor malignancy. The PPARγ protein expression in tumor tissue was dependent to the receiving of chemotherapy treatment (OR = 1.27, CI = 1.13–1.43, *P*-value < 0.0001), however to describe the exact impact of chemotherapy on the PPARγ expression pattern and also the possible effect of PPARγ over expression in the patient’s level of response to the chemotherapy, further mechanistic study is required and it cannot conclude from the present study. The expression of PPARγ at both protein and gene level showed positive predictive value to malignant tumor metastasis. Interestingly, PPARγ protein expression level significantly dependent to the tumor grade (OR = 1.22, CI = 1.10–1.36, *P*-value < 0.0001). Consistently, both PPARγ protein and gene expression was considerably effective in predicting tumor recurrence. The diagnostic value of PPAR${\upgamma }$ and the ROC curve information in different groups of primary bone tumors is revealed in Table 4. As indicated, PPARγ gene expression showed significant diagnostic value to discriminate between malignant and benign bone tumors (Cut off value < 0.11, AUC = 0.68, *P* = 0.005), also between malignant and non-cancerous bone tissues (Cut off value < 0.13, AUC = 0.80, *P* < 0.0001) as well as bone tumors and bone normal tissues (Cut off value < 0.10, AUC = 0.70, *P* < 0.0001). The diagnostic value of PPARγ protein expression level to differentiate between benign and malignant tumors (Cut off value < 10.06, AUC = 0.91, *P* < 0.0001), between benign and control groups (Cut off value < 7.41, AUC = 0.81, *P* < 0.0001), between malignant and control groups (Cut off value < 10.06, AUC = 0.96, *P* < 0.0001) and between total bone tumors and normal bone tissues (Cut off value < 7.41, AUC = 0.91, *P* < 0.0001). The specificity and sensitivity of cut off values of PPARγ protein level was more significant to discriminate different bone groups compared to PPARγ gene level.

**Table 3:** The regression of PPAR${\upgamma }$ (Logistic regression)

Dependent variable	Independent variable	OR	95% CI	*P*-Value
Malignancy (Benign Vs. Malignant)	PPAR (Gene expression)	9.83	0.02-5825.21	0.48
	PPAR (Protein level)	1.59	1.28–1.98	0.000
Chemotherapy (Negative Vs. Positive)	PPAR (Gene expression)	21.19	0.30-1497.54	0.160
	PPAR (Protein level)	1.27	1.13–1.43	0.000
Metastasis (Negative Vs. Positive)	PPAR (Gene expression)	2626.66	14.19-486194.44	0.003
	PPAR (Protein level)	1.25	1.11–1.41	0.000
Tumor grade (Low grade Vs. High grade	PPAR (Gene expression)	26.27	0.37-1873.31	0.13
	PPAR (Protein level)	1.22	1.10–1.36	0.000
Recurrence (Negative Vs. Positive)	PPAR (Gene expression)	335.71	3.58-31443.37	0.012
	PPAR (Protein level)	1.16	1.06–1.27	0.001

**Table 4:** The value of PPAR${\upgamma }$expression to discriminate between different groups of primary bone tumors (ROC curve information)

	Groups	Cutoff point	Sensitivity (%)	Specificity (%)	AUC	*P*-Value
PPAR (Gene expression)	Benign Vs. malignant	< 0.11	43	92	0.68	0.005
	Control Vs. benign	< 0.15	57	50	0.52	0.830
	Control Vs. malignant	< 0.14	65	80	0.80	< 0.0001
	Control Vs. bone tumors	< 0.10	40	90	0.71	< 0.0001
PPAR (Protein level)	Benign Vs. malignant	< 10.06	77	98	0.91	< 0.0001
	Control Vs. benign	< 7.41	80	77	0.81	< 0.0001
	Control Vs. malignant	< 10.06	90	98	0.96	< 0.0001
	Control Vs. bone tumors	< 7.41	80	92	0.91	< 0.0001

## Discussion

Primary bone tumors imposes considerable morbidity on patients and the mortality rate is still considerable, despite promising progress regarding therapeutic and diagnostic approaches [24]. The presence of heterogeneous tumors, nonspecific clinical symptoms, late diagnosis and high growth rate of bone cells are the main contributing issues that lead to failure in efficient detection and treatment [6]. Particularly current efforts are devoted to recognizing more contributing, sensitive, specific and cost-effective biomarkers for early diagnosis, effective treatment and determining molecular mediators involved in the pathophysiology of bone tumors [20, 25, 26]. Tackling the aforementioned shortages, our team aimed to clarify the expression pattern of PPARγ, as a master metabolic regulator and lipid sensor to get more insight regarding its implication in bone cancer pathogenesis. Accordingly, three types of most prevalent malignant and benign tumors, along with normal paired bone tissue corresponding to the same tumor were examined. A significant elevation of PPAR*γ* gene and protein levels of expression was observed in primary bone tumors compared to normal bone tissues in the current study. Moreover, at both PPAR*γ* protein and gene level, malignant bone tumors revealed a higher level of PPAR*γ* expression. Recent studies point to the role of PPAR*γ* in different aspects of cancer cell growth and metabolic reprogramming [27]. Consistently, it was suggested that PPAR*γ* regulate bone cell differentiation by triggering differentiation of hematopoietic stem cell (HSC) into osteoclasts and elevation of bone resorption [28]. Moreover, ligand-activated PPARγ accompanies RXRα to activate signaling pathways leading to osteoclastogenesis [15]. It was seen paradoxically that PPARγ activation suppresses mesenchymal stem cell (MSC) differentiation into osteoblasts and leads the balance to stimulate bone resorption and inhibit bone formation [29]. However, the role of PPARγ in bone cancer cell growth is intriguing and both pro-tumorigenic and anti-tumorigenic evidence is revealed. Haydon et al., showed that PPARγ agonist, Troglitazone, induced cell differentiation and apoptosis [14]; while Lucarelli et al., provided evidence that Troglitazone increased cell proliferation and inhibited apoptosis in human osteosarcoma cells [30]. In the current study, amongst malignant bone tumors, osteosarcoma tumors expressed a higher level of PPARγ gene and protein level compared to Ewing Sarcoma tumors. In justifying the difference seen between these two tumors, it should be mentioned that, in addition to the difference between these two types of malignant bone tumors at the site of tumor formation and distribution pattern in the bone, osteosarcoma tumors are frequently developed in osteoblast cells; while Ewing Sarcoma tumors triggered by chromosomal translocation and producing oncogenic fusion gene *EWS-FLI1* that leads to osteoclast activating [31]. Data regarding the relevance of PPARγ to the Ewing Sarcoma is limited and most of the results have been obtained from studies on osteosarcoma cancer cells; however, the expression pattern of PPARγ in these tumors has not been studied and most of the evidence is from the stimulation of PPARγ with various agonists and the effect on the death and proliferation of osteosarcoma cells [27]. In the current study, GCT tumors expressed lower PPARγ gene and protein levels compared to malignant bone tumors and in line with our study, Takeuchi et al., showed a patient with GCT tumor expressed PPARγ that might be related to the disease [32]. Changes in the expression level of PPARγ in other tumors with different severity have also been observed. Consistently, in the cohort of 308 patients with primary breast cancer, PPARγ was expressed in 58% of patients and its cytoplasmic expression was correlated with poor survival [33]. Also, the increased PPARγ expression was detected in malignant and high grade ovarian tumors indicating the involvement of PPARγ in ovarian tumor development [34]. Moreover, the enhanced expression of PPARγ was detected in squamous cell lung carcinoma that was associated with tumor size [35]. In agreement, in the current study the elevated PPARγ protein level was correlated with high grade, metastatic and recurrent tumors that were more prominent for osteosarcoma tumors. Although the results of in-vitro studies on osteosarcoma cells favor the role of tumor suppressor of PPARγ [36], data of the current study showed the enhanced level of PPARγ in tumors with more severity. Rationally activation of PPARγ is required for glucose and lipid metabolism to meet the high demands of energy for cancer cell growth, invasion and migration [13]. Considering the fact that mRNA expression might not end up in protein translation, therefore, the simultaneous study of PPARγ mRNA and protein in this study showed a more complete picture of the status of PPARγ. The mRNA and protein levels revealed the same expression pattern in bone tumors, but the protein results showed the greater diagnostic value and had predictive value for tumors with more severity. Notably based on our data, PPARγ protein was localized in the cytoplasm of both osteosarcoma and Ewing sarcoma tumors. Although data regarding the subcellular localization of PPARγ in bone tumors is limited, the cytoplasmic localization of PPARγ was reported in other types of tumors such as prostate cancer [37, 38]. The significance of cytoplasmic PPARγ in tumors and the mechanism of nucleocytoplasmic shuttling of PPARγ is under investigation. In accordance it was shown that S-phase kinase protein (Skp2) overexpression stimulates cytoplasmic localization of PPARγ through the MEK1-dependent pathway in breast cancer cells [39]; however, the mechanism of translocation and importance of cytoplasmic localization seemed to be tissue-dependent and is required to be clarified in other types of tumors such as bone tumors. Assessment of the PPARγ protein was measured using immunohistochemistry in the current study that the important advantage of which is the possibility of determining the location of the protein in the cell, however this study was limited in terms of protein analysis with another technique such as western blotting. Although both techniques are based on specific antigen-antibody interaction, western blot technique is advantageous in that it has proven to be more quantitative and the generated signals are proportional to the amount of the protein [40]. Accordingly, exploiting of the PPARγ protein by other techniques such as western blot is suggested in future studies to obtain more robust data. Our preliminary study was conducted using a relatively small sample size (due to the bone tumor incidence in our region); however further studies with more samples are suggested to confirm the diagnostic potential of PPARγ in bone tumors.

## Conclusions

In conclusion, the PPARγ gene and protein revealed different expression patterns in primary bone tumors with a significantly elevated level of expression in malignant tumors with higher degrees of deterioration. Our data provide insights into the efficiency of PPARγ gene and protein expression level to discriminate between primary bone tumor and healthy bone tissue; however, the PPARγ relevance to the chemotherapy outcome and response to treatment need to be validated by future studies. Also, determining the prognostic value of PPARγ in patients with varying degrees of primary bone tumor severity and investigate PPARγ protein with more detailed is suggested by further evaluations.

## Data Availability

All data generated or analyzed during this study are included in this published article.

## Abbreviations

PPARs: Peroxisome proliferator-activated receptors
VEGF-A: Vascular endothelial growth factors A
ROS: Reactive oxygen species
RXR: Retinoid X receptor
GCT: Giant Cell Tumors
FBS: Fasting Blood Sugar
OCT: Optimal Cutting Temperature
DAB: 3′-Diaminobenzidine
ROC: Receiver operating characteristic
std: Standard deviation
HSC: Hematopoietic stem cell
AUC: Area under the curve
MSC: Mesenchymal stem cell
